# International guidelines for the management of healthcare workers living with HIV: a systematic comparative review

**DOI:** 10.3205/dgkh000643

**Published:** 2026-03-20

**Authors:** Roland Diel, Albert Nienhaus

**Affiliations:** 1 Institute of Epidemiology, University Medical Hospital Schleswig-Holstein, Kiel, Germany; 2 Institution for Statutory Accident Insurance and Prevention in the Health and Welfare Services (BGW), Hamburg, Germany; 3 Competence Center for Epidemiology and Health Services Research for Healthcare Professionals (CVcare), Institute for Health Services Research in Dermatology and Nursing (IVDP), University Medical Center Hamburg-Eppendorf (UKE), Hamburg, Germany

**Keywords:** HIV, healthcare workers, exposure-prone procedures, viral suppression, viral load threshold, clinical practice guidelines

## Abstract

**Background:**

Despite strong evidence that sustained HIV viral suppression eliminates transmission risk (“undetectable equals untransmittable”, U=U), national approaches to managing healthcare workers (HCWs) living with HIV remain heterogeneous. Because such cases are rare but ethically and legally sensitive, clear guidance is needed to ensure consistent patient safety standards while avoiding unnecessary restrictions and stigma.

**Methods:**

A systematic comparative review of international guidance on the management of HCWs living with HIV was performed. A broad PubMed search identified peer-reviewed guideline-type publications, supplemented by a targeted manual search of official websites of public health authorities, occupational health agencies, and advisory bodies across European Union (EU) countries, Anglo-American countries (UK, Ireland, USA, Canada, Australia), and Japan. Identified documents were extracted and compared using a predefined framework: viral load threshold, definition of suppression, monitoring frequency (initial and after stable suppression), handling of viral load “blips,” reinstatement to exposure-prone procedures (EPPs), and oversight structure. Information on the need for patient notification and look-back procedures was also recorded.

**Results:**

The PubMed search yielded 2,851 records, but only three peer-reviewed recommendations met the inclusion criteria: the updated guidance of the Society for Healthcare Epidemiology of America (SHEA), USA; the recommendation of the German Association for the Control of Viral Diseases (DVV) and Society for Virology (GfV), Germany, and the consensus of the Japan Society for Occupational Health’s Research Group on Occupational Health for Health Care Workers. The manual search identified additional national guidance in the UK, Ireland, Canada, Australia, and several EU member states (including Switzerland, Austria, Denmark, the Netherlands, France, and Spain). Three major regulatory patterns emerged. First, multiple countries employ viral-load–based clearance for exposure-prone procedures (EPPs), most commonly using <200 copies/mL (UK, USA, Australia, Spain), while Germany applies the most stringent threshold (=50 copies/mL) with detailed “blip” rules and quarterly monitoring. Second, some jurisdictions adopt non-numerical, autonomy-oriented frameworks: the Netherlands relies on the treating physician’s responsibility under U=U without mandatory panels or reporting, while Denmark uses a universal infection-control approach that does not operationalize HIV status for practice restrictions. Japan similarly lacks numeric thresholds and formal oversight, delegating decisions locally while citing UK/US models as references. Third, Switzerland represents a conservative, procedure-heavy model without explicit thresholds, emphasizing external committee involvement and discretionary decision-making. Many other EU states show regulatory silence or only vague discretionary authority without HIV-specific operationalization.

**Conclusion:**

International guidance has converged on viral suppression as the decisive determinant of patient safety, but implementation differs markedly in thresholds, monitoring intensity, and governance. Most guidelines specifying a numerical criterion align on <200 copies/mL as the operative standard for eligibility to perform EPPs, effectively establishing an international benchmark. Greater harmonization toward transparent, evidence-based standards may reduce regulatory inconsistency and legal uncertainty while preserving patient protection. Future research should assess whether threshold-based models with defined monitoring and reinstatement mechanisms provide a more reliable and evaluable framework for ensuring patient safety than non-numerical approaches.

## Zusammenfassung

**Hintergrund:**

Trotz wissenschaftlicher Evidenz, dass eine dauerhaft supprimierte HIV-Viruslast das Übertragungsrisiko eliminiert (“undetectable equals untransmittable” U=U, „nicht nachweisbar bedeutet nicht übertragbar“), unterscheiden sich die nationalen Ansätze zum Management von mit HIV infiziertem medizinischem Personal (MP) weiterhin erheblich. Da solche Fälle selten, zugleich jedoch ethisch und rechtlich sensibel sind, besteht Bedarf an klaren Leitlinien, um die Patientensicherheit zu gewährleisten und gleichzeitig unnötige Einschränkungen und Stigmatisierungen zu vermeiden.

**Methode:**

Es wurde ein systematisches Review internationaler Empfehlungen zum Management von mit HIV infiziertem MP durchgeführt. Eine breit angelegte PubMed-Suche identifizierte peer-reviewte Leitlinienpublikationen, ergänzt durch eine gezielte manuelle Recherche auf den Websiten von Gesundheitsbehörden, arbeitsmedizinischen Institutionen und Beratungsgremien in EU-Mitgliedstaaten, ausgewählten anglo-amerikanischen Ländern (Vereinigtes Königreich, Irland, USA, Kanada, Australien) sowie Japan. Die identifizierten Dokumente wurden anhand folgender Kriterien ausgewertet und verglichen: Viruslast-Schwellenwert, Definition der Suppression, Überwachungsintervalle (initial und nach stabiler Suppression), Umgang mit vorübergehenden Viruslastanstiegen („Blips“), Wiederzulassung zu expositionsanfälligen Tätigkeiten (EPPs) sowie Aufsichts- und Entscheidungsstrukturen. Angaben zur Notwendigkeit von Patientenbenachrichtigungen und Look-back-Untersuchungen wurden ergänzend erfasst.

**Ergebnisse:**

Die PubMed-Suche ergab 2 851 Treffer, von denen lediglich drei peer-reviewte Empfehlungen die Einschlusskriterien erfüllten: die aktualisierte Empfehlung der Society for Healthcare Epidemiology of America (SHEA), USA; die Empfehlung der Deutschen Vereinigung zur Bekämpfung der Viruskrankheiten (DVV) und der Gesellschaft für Virologie (GfV), Deutschland, und die Konsensusempfehlung der Japan Society for Occupational Health’s Research Group on Occupational Health for Health Care Workers. Die manuelle Recherche identifizierte zusätzliche nationale Leitlinien im Vereinigten Königreich, Irland, Kanada, Australien sowie in mehreren EU-Staaten (u. a. Schweiz, Österreich, Dänemark, Niederlande, Frankreich, Spanien). Es lassen sich drei regulatorische Grundmuster unterscheiden: Mehrere Länder wenden viruslastbasierte Freigabekriterien für EPPs an, meist mit einem Schwellenwert von <200 Kopien/ml (UK, USA/SHEA, Australien, Spanien), während Deutschland den strengsten Grenzwert (=50 Kopien/ml) mit detaillierten „Blip“-Regeln und vierteljährlichem Monitoring festlegt. Andere Staaten verfolgen nicht-numerische, autonomiebetonte Ansätze. Die Niederlande stützen sich auf die Verantwortung der behandelnden Ärztinnen und Ärzte ohne verpflichtende Expertengremien. Dänemark nutzt ein universelles Infektionsschutzmodell ohne HIV-spezifische Praxisrestriktionen. Japan verzichtet ebenfalls auf numerische Schwellenwerte und formale Aufsicht, verweist jedoch auf britische und US-amerikanische Modelle. Hiervon abweichend verfolgt die Schweiz einen stark prozeduralen Ansatz ohne explizite Grenzwerte, aber mit der Fokussierung auf externe Gremien und Ermessensentscheidungen. In vielen weiteren EU-Staaten finden sich keine Empfehlungen oder lediglich unspezifizierte Ermessensbefugnisse ohne HIV-spezifische Operationalisierung.

**Schlussfolgerungen:**

International besteht Einigkeit darüber, dass eine supprimierte Viruslast den entscheidenden Maßstab für die Patientensicherheit darstellt. Die Umsetzung dieser Erkenntnis variiert jedoch erheblich hinsichtlich Schwellenwerten, Überwachungsintensität und Governance-Strukturen. Die meisten Leitlinien, die ein numerisches Kriterium festlegen, orientieren sich an einem Grenzwert von <200 Kopien/mL als maßgeblichem Standard für die Durchführung expositionsanfälliger Eingriffe und etablieren damit faktisch einen internationalen Referenzwert. Eine stärkere Harmonisierung hin zu transparenten, evidenzbasierten Standards könnte regulatorische Inkonsistenzen und rechtliche Unsicherheiten verringern und zugleich ein hohes Maß an Patientenschutz gewährleisten. Zukünftige Forschung sollte prüfen, ob schwellenwertbasierte Modelle mit klar definierten Überwachungs- und Wiederzulassungsregelungen in der Praxis eine verlässlichere und besser evaluierbare Grundlage für Patientensicherheit bieten als nicht-numerische Ansätze.

## Introduction

Since the introduction of highly active antiretroviral therapy (HAART) in the mid-1990s, HIV infection has been transformed from a fatal disease into a manageable chronic condition. Landmark randomized clinical trials demonstrated that combination antiretroviral therapy, including protease inhibitors, could achieve sustained suppression of viral replication, dramatically reducing AIDS-related morbidity and mortality and establishing viral load as the central therapeutic and prognostic marker of HIV infection [1], [2]. These advances fundamentally altered both the clinical course of HIV and its epidemiological implications.

Subsequent large-scale randomized trials and prospective cohort studies have shown that sustained viral suppression not only prevents disease progression but also effectively eliminates the risk of HIV transmission. The preventive effect of antiretroviral therapy was first demonstrated in the HPTN 052 trial, which showed a near-complete prevention of sexual transmission when treatment was initiated early [3], [4]. These findings were later confirmed in large European cohort studies, including the PARTNER and PARTNER2 studies, which observed no linked transmissions among thousands of condomless sexual acts when the HIV-positive partner maintained viral suppression [5]. Together, this body of evidence gave rise to the principle now widely recognized as Undetectable = Untransmittable (U=U), a concept formally articulated and disseminated through an international consensus statement and subsequently endorsed by major public health authorities and professional societies [6].

In parallel with these therapeutic advances, the epidemiology of HIV among healthcare workers (HCWs) in high-income countries has changed profoundly. Since the HAART era, HIV prevalence among HCWs has remained consistently low, and documented cases of provider-to-patient transmission have become exceedingly rare. Surveillance data and systematic reviews indicate that occupationally acquired HIV infection among HCWs is now an exceptional event, and no confirmed cases of transmission from virally suppressed HCWs to patients have been documented in modern healthcare settings [7], [8], [9]. Outside sub-Saharan Africa, where the burden of HIV remains substantially higher, HIV infection among HCWs represents a marginal occupational health issue in numerical terms.

At first glance, this epidemiological reality might suggest that specific guidance on the professional practice of HIV-infected HCWs is no longer necessary. However, low prevalence does not equate to zero risk, nor does it eliminate the need for clear regulatory frameworks. Healthcare systems must be prepared not only for common scenarios but also for rare and highly sensitive ones, particularly where patient safety, professional rights, occupational health, and public trust intersect. The management of HCWs living with HIV represents precisely such a scenario: infrequent in occurrence, yet potentially profound in its ethical, legal, and professional implications.

In the absence of explicit guidance, decisions regarding fitness to practice, exposure-prone procedures (EPPs), viral-load monitoring, and patient notification are often made ad hoc at institutional or regional level. Such informal decision-making risks inconsistency, legal uncertainty, and unnecessary restrictions on affected HCWs, while offering no demonstrable improvement in patient safety. Conversely, overly restrictive or outdated policies may perpetuate stigma, discourage disclosure, and undermine occupational health principles that are otherwise grounded in evidence-based risk assessment.

Against this background, a systematic comparison of national and professional guidelines on the management of HIV-infected HCWs remains both relevant and necessary. Such an analysis allows identification of common principles, critical divergences, and regulatory gaps, and helps to distinguish differences driven by governance structures from those reflecting genuine differences in risk tolerance or ethical priorities. Importantly, it also provides a framework for assessing whether existing policies adequately balance patient safety with the rights, dignity, and professional autonomy of HCWs living with HIV. The present review therefore aims to systematically compare international guidelines and recommendations addressing the management of HIV-infected HCWs, with particular attention to EPPs, viral-load thresholds, monitoring requirements, oversight mechanisms, and responses to loss of viral suppression.

## Method

### Search strategy and identification of national guidelines

A structured literature search was conducted to identify guideline-type publications addressing the management of HCWs living with HIV. The method comprised two complementary components: (1) a systematic database search to identify peer-reviewed recommendations and consensus statements, and (2) a targeted search for national guidelines and policy documents issued by public health authorities or professional bodies.

### Database search

First, a structured search was conducted in PubMed without language restrictions or restrictions on the year of publication. The search strategy was designed to maximize sensitivity and ensure retrieval of all relevant peer-reviewed guidance documents authored by expert groups. To this end, the search combined three conceptual domains: HIV infection, healthcare personnel, and guideline-related publication types. The final PubMed search strategy was as follows:

(HIV[tiab] OR "HIV Infections"[Mesh] OR "human immunodeficiency virus"[tiab])

AND

("health personnel"[Mesh] OR "healthcare worker*"[tiab] OR "health care worker*"[tiab] OR "medical staff"[tiab])

AND

(guideline[pt] OR practice guideline[pt] OR guideline*[tiab] OR recommendation*[tiab] OR policy*[tiab] OR "position statement"[tiab])

Truncation (*) was applied to free-text terms to capture singular and plural forms (e.g. guideline/guidelines, recommendation/recommendations, worker/workers).

### Identification of national guidelines and policy documents

In addition to the database search, a targeted manual search was conducted to identify national guidelines, recommendations, and policy documents not consistently indexed in bibliographic databases. This component focused on countries of the EU, Anglo-American countries (United Kingdom, Ireland, United States, Canada, Australia), and Japan.

For each country, the official websites of ministries of health, national public health authorities, occupational health agencies, and national advisory bodies were systematically screened. Where necessary, targeted web searches were performed using Google, combining HIV-related terms with healthcare worker–specific terminology and country-specific government domains. Examples of search queries included:

- “HIV healthcare worker guideline site:gov.uk”
- “HIV infected health worker recommendation site:gov.au”
- “exposure-prone procedures HIV guideline site:go.jp”
- “HIV health care worker site:gov.ie”.

Recommendations issued by national health authorities or recognized professional associations were eligible for inclusion. Each identified source was documented by country, issuing institution, year of publication, and full internet address.

Where national recommendations were available only in the original language, translations into English were produced using the DeepL translation tool. 

### Structure of the results and comparative framework (aligned with table headings)

Following the identification of relevant national and professional guidance documents, the results are presented in two complementary formats. First, each guideline or recommendation is described individually in narrative form to reflect its regulatory context, scope, and underlying conceptual approach. 

Second, where sufficient information was available, key elements of the identified guidelines were extracted and compared using a predefined set of criteria and summarized in a comparative table. The comparative framework was developed *a priori* to capture operationally relevant aspects of guideline implementation and to facilitate cross-country comparison.

The predefined comparison criteria correspond directly to the table columns and included: viral load threshold, definition of viral suppression, initial monitoring frequency, monitoring after stable suppression, handling of viral-load “blips”, reinstatement to EPPs and oversight structure. Where explicitly addressed by the guidance, additional aspects such as patient notification policies and look-back investigations were also extracted and reported.

Together, the narrative descriptions and the comparative table provide a structured overview of similarities and differences across international approaches to the management of HCWs living with HIV.

## Results

The PubMed search yielded 2,851 records. Of these, only three publications fulfilled the predefined inclusion criteria for guideline-type publications explicitly addressing the management of HCWs living with HIV. These comprised the updated guidance of the Society for Healthcare Epidemiology of America (SHEA), USA [10], the recommendation of the German Association for the Control of Viral Diseases (DVV) and the Society for Virology (GfV), Germany [11], and the consensus of the Japan Society for Occupational Health’s Research Group on Occupational Health for Health Care Workers [12].

By contrast, the targeted manual search of official websites of national health authorities, and public health institutions identified national guidance documents or ministerial decrees in all of the predefined Anglo-American countries and Japan, as well as in the EU member states Germany, Austria, Denmark, Netherlands, France, Spain, and Hungary and the non EU member Switzerland. For all remaining EU member states, no publicly available national guidance documents specifically addressing the management of HCWs living with HIV could be identified. However, two distinct regulatory patterns emerged. In most countries, HIV-infected HCWs are not mentioned at all in national regulations or guidance documents (“regulatory silence”). By contrast, in Slovakia and Italy, general public health legislation contains vague enabling provisions that allow for professional restrictions in the presence of a concrete transmission risk, without specifying HIV-specific criteria, operational thresholds, or structured decision-making processes. 

### Countries with explicit HIV-specific HCW guidelines

The detailed description reflecting differernt countries with explicit HIV-specific HCW guidelines are displayed in Table 1 (Tab. 1) and Table 2 (Tab. 2).

#### United Kingdom

The UK Advisory Panel (UKAP) 2024 [13] framework translates current evidence into a detailed regulatory structure. An initial clearance for exposure-prone work of an HIV-infected HCW requires two identified and validated samples (IVS), taken at least 12 weeks apart and both showing viral loads <200 copies/mL. Thereafter, ongoing monitoring is compulsory (remains permanent) every 12 weeks (quarterly), and missing this time window automatically suspends the HCW from performing EPPs until two new IVS, 12 weeks apart, confirm suppression. 

Viral loads between 200 and 1,000 copies per mL necessitate immediate retesting after 10 days. If the result remains >200 copies/mL, EPPs must cease until two consecutive IVS ≥12 weeks apart confirm suppression. For results ≥1,000 copies/mL, EPPs must cease immediately. A repeat test is required after 10 days, and if confirmed, a full risk assessment is initiated, potentially including a Patient Notification Exercise (PNE). These measures are overseen by accredited occupational medicine specialists, in consultation with UKAP. Although the national Occupational Health Register (UKAP-OHR) was decommissioned in 2024, local record-keeping remains obligatory. 

#### United States of America

Also the SHEA 2022 framework [10], upholds the principle that a HCW with a sustained viral load below 200 copies/mL poses no measurable risk and should not face categorical restrictions on professional practice. Monitoring every six months is generally considered standard. Decisions to restrict clinical duties are made case by case, guided by contextual factors rather than automatic rules. A defining feature of the updated SHEA guidance is its explicit acceptance of transient “blips” — minor, short-term viral rebounds that do not signify treatment failure or an increased risk of transmission. SHEA emphasizes that no clinical or occupational decision should be based on a single viral-load result, advocating instead for repeat testing and contextual evaluation by the Expert Review Panel. 

#### Japan

Japan does not have a binding national government guideline for the management of HCWs living with HIV. In the absence of formal regulation, the 2017 consensus statement of the Japan Society for Occupational Health (JSOH) functions as the country’s most authoritative source of guidance [12]. A central feature of the Japanese framework is the absence of any numerical viral-load threshold. Unlike the UK and USA, which explicitly define suppression as <200 copies/mL, Japan sets no quantitative boundary for determining whether an HIV-positive HCW may perform EPPs. The consensus acknowledges that transmission risk is extremely low when HIV viral load is well controlled, yet it does not translate this scientific principle into specific operational rules. Instead, assessing transmission risk is delegated to occupational health professionals, who are instructed to evaluate each case individually and modify clinical duties if necessary.

The document neither bans EPP participation nor provides formal criteria for allowing it. Decisions about task modification, redeployment, or continued EPP practice must be made locally.

Although Japan does not adopt viral-load thresholds or EPP algorithms, it notably includes explicit references to the UK and US systems. The consensus cites both the SHEA guideline [10] and the UK Department of Health/UKAP framework [13] as international models and even reproduces a comparison table summarizing their viral-load-based criteria. However, these references are treated as illustrative examples, not as policies to be incorporated into Japanese practice. The document instead reiterates the centrality of case-by-case assessment.

The Japanese approach also lacks formal oversight mechanisms comparable to UKAP-OHR or institutional Oversight Panels in the US. Instead of establishing national or regional expert review committees, Japan relies on ad hoc, institution-level consultation among occupational health physicians, infectious disease specialists, and administrators. Monitoring intervals are not defined; the consensus simply states that HCWs should maintain appropriate treatment and follow-up with their HIV physician.

In summary, the JSOH 2017 consensus represents a non-numerical, decentralized, rights-oriented framework that delegates all decision-making to local occupational health teams, emphasizes confidentiality and non-discrimination, and encourages institutions to use international guidelines as reference points without adopting their explicit rules. Japan therefore occupies an intermediate regulatory position: more progressive and ethically grounded than prohibition-based systems such as Ireland’s, yet less operationally modern than viral-load-driven clearance frameworks used in other high-income countries.

#### Spain

The 2023 Spanish national recommendations [14], issued by the Spanish Ministry of Health (Ministerio de Sanidad) also state that HCWs with HIV infection who have an undetectable viral load (<200 copies/mL), either because they are receiving antiretroviral treatment or because they are elite controllers, should not be subject to any restrictions. If a worker has a detectable viral load, either due to a new diagnosis or virological failure, they are temporarily restricted from performing EPP until viral suppression is re-established. The protocol for viral-load monitoring is quantitative and detailed: while the viral load remains detectable (≥200 copies/mL), testing is required every three months; after suppression (<200 copies/mL), the same interval continues until two consecutive undetectable results are achieved, each at least three months apart. Once viral suppression is stable, testing may be extended to every six months. Before resuming EPPs, two separate undetectable results are required, each taken at least three months apart with a detection limit below 200 copies/mL. HIV-positive professionals have no legal obligation to disclose their status to patients or employers and maintain full confidentiality regarding their condition. Institutions are encouraged to establish an Evaluation Commission (Comisión de Evaluación) to support case management on a multidisciplinary basis. In the event of potential patient exposure to an infected worker’s blood, the patient must be informed and offered post-exposure prophylaxis (PEP), while the identity and serostatus of the healthcare worker remain confidential. 

#### Germany

The German national guideline jointly issued by the German Association for the Control of Virus Diseases (DVV) and the Society for Virology (GfV) in 2012 [11] combines strict requirements for viral suppression with a clearly defined procedure for handling transient viral ‘blips’. HIV-positive HCWs are permitted to perform all medical duties, including EPPs, provided their viral load is ≤50 copies/mL as very stringent quantitative threshold. Thus, Germany enforces one of the strictest thresholds worldwide. Viral load must be routinely monitored every three months. The results, though confidential, must be shared with the occupational physician, who ensures compliance with the 50-copy threshold. If this limit is exceeded, the case must be presented to an expert panel that decides on temporary restrictions or reinstatement once viral suppression is re-established.

Any transient increase between 51 and 500 copies per mL is defined as a “blip” and tolerated provided that it resolves within 14 days on repeat testing to determine whether this represents a temporary fluctuation or persistent viremia. During this observation period, HCWs may continue their professional duties provided that previous viral load measurements were stable (≤50 copies/mL). In the case of a confirmed transient blip (≤500 copies/mL, resolving within about 14 days), this is classified as a harmless fluctuation, and the person may continue to work without restriction. If there is persistent low-level viremia (51–500 copies/mL) lasting more than 14 days or if there is any increase exceeding 500 copies/mL the person is excluded from performing EEPs or other invasive tasks. Reinstatement is only granted once viral load has returned to ≤50 copies/mL and remains stable. The German document therefore provides unambiguous operational thresholds, fixed testing intervals, and clear stop-and-restart criteria.

#### France

The French national recommendations issued by the Haut Conseil de la Santé Publique (HCSP) in 2011 [15] states that when the viral load is undetectable according to a sensitive assay, the HCW may perform EPP without restriction. No procedural exclusion is required provided that standard precautions are rigorously applied and regular viral-load monitoring is maintained; however, the guidance deliberately refrains from specifying the monitoring interval. The HCSP also introduces a non-binding, indicative threshold to characterize “high viral load”: >200 copies/mL for HIV (alongside >10^4^ IU/mL for HBV/HCV). Although explicitly described as a “very rough” guide, in such cases, corrective antiretroviral management and, where appropriate, temporary modification of duties are recommended until viral suppression is re-established. That means that in such cases the guidance merely recommends specialist consultation and, where appropriate, antiretroviral therapy to reduce viremia before reassessing fitness for practice. Thus, a confidential, case-by-case medical assessment is recommended when an infected HCW performs EPPs. The treating physician may, if necessary, consult the national expert commission to obtain advice on whether the healthcare worker may continue such duties, but this referral is voluntary and advisory only. The commission’s role carries no regulatory authority, and HCWs are not required to inform employers, patients, or health authorities of their infection or viral-load result; patient notification is only contemplated after a documented exposure incident, accompanied by appropriate PEP. 

The 2011 guidance establishes no statutory or formal reporting obligation for HIV-infected HCWs. Instead, it recommends a confidential, case-by-case medical assessment when an infected HCW performs EPPs. The treating physician may, if necessary, consult the national expert commission (‘commission d’experts’) to obtain advice on whether the HCW may continue performing EPPs. This referral is voluntary, not mandatory. The commission’s role is strictly advisory and carries no disciplinary or regulatory authority. HCWs are not required to inform employers, patients, or health authorities of their infection or viral-load results. Disclosure is only envisaged if a documented exposure incident occurs that requires post-exposure prophylaxis for the patient. Thus, France operates on a principle of medical discretion and voluntary consultation rather than a legal or administrative duty to report.

#### Switzerland

The Swiss national guideline issued by the Federal Office of Public Health (Bundesamt für Gesundheit, BAG) in 2011 and updated in 2013 [16] acknowledges that HIV infection does not automatically preclude performing EPPs its approach remains procedurally complex and lacking in clarity The document does not specify any quantitative viral load threshold — such as 200 or 1,000 copies per mL — below which exposure-prone procedures are permitted. There is no prescribed schedule for regular viral-load testing, and antiretroviral therapy is recommended only as a risk-reduction measure rather than as a mandatory condition for clinical practice. A key passage in Section 4.7 of the guideline states that persons infected with HIV should consult a specialist to clarify whether treatment is indicated, adding that reduction of viraemia “can help” to reduce transmission risk. This wording presents treatment as an optional intervention rather than a requirement for continued practice. Consequently, Switzerland never adopted a viral-load-based fitness-to-practice threshold and has not incorporated the U=U principle into its regulatory framework.

The guideline establishes a multi-step procedural process for HIV-infected HCWs who perform EPPs. It involves an initial consultation with a specialist, typically an infectious disease physician, followed by an occupational evaluation conducted by the institutional or cantonal occupational health authority, and the involvement of an advisory expert group to issue recommendations regarding the worker’s continued clinical activity. Although the document suggests that the involvement of the expert group “should be recommended,” in practice this step has become effectively mandatory, especially for legal protection of both the healthcare worker and the employer. The group’s role is formally advisory, but its recommendations carry decisive weight. The final administrative authority lies with the cantonal physician, who makes the ultimate decision based on the advisory expert group’s opinion. Thus, this arrangement transfers decision-making power from the treating physician and the HCW to an external committee, creating a system that prioritizes institutional caution over individual medical judgment.

Perhaps the most revealing aspect of the BAG guidance is found in the statement that “vocational retraining should be discussed.” Appearing immediately after the note that EPPs are “not contraindicated,” this phrasing sends a contradictory signal. While the text formally permits continued clinical work, it simultaneously introduces career change as a recommended alternative, generating psychological and professional pressure for infected HCWs to withdraw voluntarily from invasive procedures. 

#### The Netherlands

The Netherlands has adopted a distinctively liberal and trust-based regulatory approach to the management of HCWs living with HIV by a dedicated, HIV-specific guidance document entitled “Preventie van transmissie van hiv door risicovormend medisch personeel” [17].

The current Dutch guidance does not introduce numerical viral-load thresholds or prescriptive management algorithms. Instead, it establishes a principles-based framework grounded in current scientific evidence and professional ethics, explicitly rejecting automatic restrictions on clinical practice based solely on HIV serostatus. Assessment of fitness to practice, including participation in exposure-prone procedures, is entrusted to the treating physician, typically an HIV specialist, who bears both the medical and ethical responsibility for ensuring that effective antiretroviral therapy is in place and that viral suppression is achieved and maintained.

When viral suppression is achieved, HCWs may continue all clinical activities without mandatory reporting to public authorities, without inclusion in a national registry, and without routine disclosure to employers, licensing authorities, or patients. External oversight bodies, formal expert review panels, or predefined reinstatement procedures are not foreseen. Taken together, the Dutch approach represents a coherent, non-numerical implementation of the U=U principle that builds upon the earlier LCI framework and relies on professional responsibility rather than regulatory enforcement.

#### Denmark

Also the Danish national guideline ‘Vejledning om HIV, hepatitis B og C virus’ (Danish Health Authority, 2013 [18]) establishes no quantitative viral load thresholds, no mandatory monitoring intervals, and no formal expert committee for individual case review. 

In Denmark, healthcare professionals infected with HIV, hepatitis B, or hepatitis C are not subject to professional restrictions, provided that standard infection control measures are applied. The prevention of occupational transmission relies entirely on universal precautions (safe handling of sharps, gloves, protective equipment, and hygiene routines) rather than on the virological status of the healthcare worker. The guideline explicitly states that there are no general restrictions for persons living with HIV in healthcare or other occupational settings. All professional activities, including EPPs, may be performed without limitation when appropriate hygiene and safety protocols are observed. 

The Danish model operates without a centralized expert panel or clearance process. Decisions are handled locally, often in collaboration between occupational health services and the treating physician. The concept of transient viral ‘blips’ is not defined, since viral load monitoring plays no role in determining professional eligibility. This decentralized approach reflects the Danish emphasis on trust, equality, and self-responsibility rather than bureaucratic control. Confidentiality and data protection are explicitly safeguarded. Disclosure of infection status to employers is prohibited, and the medical confidentiality of HIV, HBV, and HCV-positive personnel is strongly protected. Mandatory notification of infections exists only for epidemiological surveillance through the Statens Serum Institute, without implications for employment. 

In summary, the Danish guideline neither defines viral load limits nor requires expert committee approval. The system’s success depends entirely on professional integrity and an advanced ethical culture — conditions that may not be equally robust everywhere. 

While both the Netherlands [17] and Denmark [18] apply non-numerical and non-restrictive approaches to the management of HCWs living with HIV, the underlying regulatory logic differs. In the Netherlands, decisions regarding fitness to practice, including participation in exposure-prone procedures, are delegated to the treating physician, who is expected to exercise professional and ethical judgement based on individual clinical circumstances, treatment adherence, and sustained viral suppression, without reliance on prescriptive regulatory thresholds. In contrast, the Danish model adopts a universalist infection-control approach in which HIV status is considered irrelevant for professional practice, provided that standard infection-control precautions are consistently applied, thereby eliminating the need for individualized medical or ethical case assessment.

#### Austria

The joint German-Austrian guideline [19] explicitly builds on the German DVV/GfV recommendations in 2012 [11] concerning HIV-infected HCWs and the prevention of transmission. It stipulates that HIV infected healthcare professionals perform all medical procedures, provided that their plasma viral load remains at or below 50 copies/mL, that their condition is monitored at least quarterly, and that they are under the care of an HIV specialist. Austria does not operate with its own viral-load thresholds or maintain a separate expert commission for such cases.

#### Australia

Australia has adopted one of the most comprehensive and operationalized national frameworks worldwide for the management of HCWs living with HIV [20]. The Australian guideline, issued by the Communicable Diseases Network Australia (CDNA), applies a strictly algorithm-based, viral-load-driven approach to HCWs performing EPPs. Eligibility to perform EPPs requires sustained viral suppression below 200 copies/mL, supervision by an HIV specialist, and mandatory three-monthly viral load monitoring.

A distinctive feature of the Australian framework is its detailed and graded management of detectable viraemia. The guideline explicitly differentiates low-level viraemia (“blips”) from clinically relevant viral rebound and defines short, predefined verification intervals. Viral load results between 50 and 200 copies/mL do not automatically require cessation of EPPs; instead, a repeat viral load test after 10 days may be performed to confirm whether the elevation is transient. If repeat testing confirms persistence within this range, EPPs may continue, with emphasis on treatment adherence and ongoing routine monitoring. When viral load rises to 201–999 copies/mL, EPPs must be temporarily suspended, and a repeat test after 10 days is mandatory. If viral suppression below 200 copies/mL is not re-established, EPPs remain prohibited until suppression is confirmed in two consecutive tests at least three months apart.

A viral load of ≥1,000 copies/mL mandates immediate cessation of EPPs and triggers a formal expert risk assessment involving public health authorities. While routine disclosure of HIV status to patients is not required when virological criteria are met, the guideline explicitly requires structured consideration of a look-back investigation at this threshold, particularly in the presence of procedural breaches or other risk-enhancing factors. Patient notification is not automatic but must be formally assessed as part of the expert review process.

In comparison with the United Kingdom [13], the Australian framework is closely aligned in principle but differs in operational intensity. UKAP guidance likewise permits HIV-infected HCWs to perform EPPs under defined conditions, uses viral load as the central determinant of transmission risk, endorses a <200 copies/mL threshold, and rejects routine patient disclosure under viral suppression. However, UK guidance typically relies on six-monthly viral load monitoring and does not define numeric escalation thresholds for detectable viraemia or explicit triggers for look-back investigations. By contrast, the Australian CDNA guideline uniquely specifies graded responses to viral load fluctuations, including explicit thresholds linked to mandatory actions.

Overall, the Australian framework operationalizes the U=U principle through a highly prescriptive escalation pathway, combining frequent monitoring, rapid confirmation of viremic episodes, and clearly defined governance responses. This makes Australia the most algorithmic and precautionary implementation of a viral-load–based, U=U-aligned governance model among comparable high-income countries, without implying increased transmission risk under sustained viral suppression.

#### Canada

Canada has adopted a federal, principles-based framework for the management of HCWs living with HIV, as outlined in the 2019 guideline issued by the Public Health Agency of Canada (PHAC) [21]. The guideline explicitly addresses HIV-infected HCWs but deliberately refrains from defining numerical viral-load thresholds or prescriptive management algorithms. Instead, it emphasizes individual risk assessment, expert oversight, and strict adherence to standard infection prevention measures.

The PHAC guideline acknowledges that effective antiretroviral therapy substantially reduces the risk of HIV transmission and permits continued clinical practice, including invasive activities, where transmission risk is assessed as negligible. Decisions regarding work restrictions, temporary modification of duties, or reinstatement to EPPs are made on a case-by-case basis through occupational health services and expert consultation, rather than through predefined viral-load cut-offs.

While the guideline avoids fixed monitoring intervals and tiered escalation pathways, it does explicitly recognize the occurrence of transient low-level viraemia. Viral-load “blips” of up to 400 copies/mL are described as potentially clinically insignificant and are not considered to indicate treatment failure or to automatically trigger practice restrictions or look-back investigations. Beyond this limited quantitative reference, management remains grounded in expert judgment rather than algorithmic triggers.

The guideline does not mandate routine disclosure of HIV status to patients and does not specify automatic criteria for retrospective patient notification. Look-back investigations are reserved for exceptional circumstances, such as significant breaches in infection prevention measures or situations in which a credible transmission risk cannot be excluded.

In contrast to the highly algorithmic Australian framework and the threshold-based approaches adopted in the United Kingdom and the United States, Canada’s federal guidance represents a non-numerical, expert-driven model that operationalizes the U=U principle through professional judgment and decentralized decision-making rather than through formalized escalation pathways.

#### Ireland

Based on a detailed review of the 2 documents available from the Irish Health Service Executive (HSE) – the 2005 policy report “The Prevention of Transmission of Blood-Borne Diseases in the Health-Care Setting” [22] and the implementing HSE HR Circular 012/2009 [23] – the regulations governing HCWs infected with HIV are unequivocal. The central and non-negotiable rule is that HCWs known to be infected with HIV are absolutely prohibited from performing EPPs. 

However, on the other side the framework emphasizes that the employing authority must make every effort to redeploy the infected HCW within the organization or provide retraining for a new role that does not involve EPPs. This process is described as “vocational rehabilitation”, focusing on retaining the individuals skills and experience within the health service. A comprehensive support package is outlined, including maintained salary, coverage of medical treatment costs, and access to a National Advisory Panel to assist with the transition.

The process for managing an identified case is clearly structured to balance patient safety with worker confidentiality. It begins with the ethical and legal obligation of the HCW to voluntarily disclose their status to an Occupational Health Physician. They must immediately cease all EPPs pending formal assessment. A Local Expert Group is then convened by the Director of Public Health to anonymously evaluate the case and recommend any necessary work restrictions. This group also determines if a “look-back” exercise to notify past patients is warranted, which for HIV is considered highly unlikely due to the extremely low transmission risk. In conclusion, the Irish regulations definitively bar HIV-positive HCWs from performing EPPs, but they are fundamentally designed to be a supportive mechanism that protects patients while preserving the careers and rights of infected staff through redeployment and retraining. This regulatory silence contrasts sharply with viral-load-based, risk-oriented approaches adopted in countries such as the United Kingdom, the United States and Australia. 

The remaining EU member states form a broad non-guideline cluster, indicating that HIV-infected HCWs have generally not been addressed as potential sources of provider-to-patient transmission at national level. Within this group, two distinct regulatory patterns can be distinguished: countries characterized by complete regulatory silence, and countries in which general legal provisions allow for discretionary restrictions without specifying HIV-specific criteria or operational frameworks.

### Countries without explicit HIV-specific HCW guidelines

#### Hungary, Slovakia, and Italy

They fall into the category of unspecified discretionary authority, albeit for different historical and legal reasons.

Hungary historically applied a restrictive, prohibition-based approach to HCWs living with HIV. Under Decree 18/1998 (X.6.) Népjóléti Minisztérium (the Minister of Health) [24], which regulated health requirements for HCWs, HIV infection was explicitly listed as a condition incompatible with certain healthcare activities. The decree mandated compulsory medical screening of HCWs, and a confirmed HIV diagnosis resulted in a declaration of unfitness for professional activities involving a risk of transmission. In practice, this amounted to a de facto occupational ban for HIV-infected HCWs in surgical, dental, and other invasive medical specialties.

With the adoption of Decree 50/2013 NM [25] which replaced earlier regulations on hygiene, patient safety, and epidemiological conditions in healthcare institutions, these restrictive provisions were not explicitly repealed, nor were they replaced by a modern, HIV-specific framework for the management of infected HCWs. As a result, Hungary currently occupies an ambiguous regulatory position in which historic exclusionary rules formally persist, while no updated guidance reflecting contemporary evidence on viral suppression or the U=U principle has been introduced. This situation creates a regulatory vacuum in which outdated prohibitions remain embedded in legacy legislation without formal revision or replacement by risk-based, evidence-informed policies.

In Slovakia, the regulatory framework relevant to HCWs living with HIV is primarily defined by Act No. 355/2007 Coll. on the Protection, Promotion and Development of Public Health [26]. This legislation establishes a general legal basis for public health authorities to intervene in cases of infectious diseases, including HIV, and allows for restrictions on professional activities only where a concrete and demonstrable transmission risk is identified. However, the Act does not contain HIV-specific provisions addressing the professional practice of healthcare workers. In particular, it does not mandate compulsory HIV testing of healthcare personnel, does not establish routine screening programes, and does not impose automatic or categorical professional bans on HIV-positive HCWs. Instead, it provides a broad public health framework that permits case-by-case intervention without defining criteria for exposure-prone procedures, viral-load thresholds, or structured oversight mechanisms. No publicly available national regulations or guidelines supplementing Act No. 355/2007 with HIV-specific provisions for HCWs could be identified, placing Slovakia at the interface between regulatory silence and undefined discretionary authority.

In Italy, the *Linee guida sull’utilizzo dei farmaci antiretrovirali e sulla gestione diagnostico-clinica delle persone con infezione da HIV-1* [27] focus exclusively on the clinical management of people living with HIV, including antiretroviral therapy, virological suppression, and long-term care. These guidelines do not address occupational practice, exposure-prone procedures, fitness for work, or transmission risks associated with HIV-infected healthcare workers, nor do they consider the professional role of the individual as a HCW.

Similarly, Italian occupational health and biosafety legislation, most notably Legislative Decree No. 81/2008, conceptualizes HIV exclusively as an occupational hazard to which HCWs may be exposed [28]. The regulatory emphasis is placed on protecting HCWs from biological risks through preventive measures such as standard precautions, post-exposure prophylaxis, and workplace safety regulations. These legal provisions do not consider infected HCWs as potential sources of transmission and do not introduce mechanisms for practice restrictions, viral-load-based risk assessment, expert review panels, or clearance procedures for exposure-prone practice. Italy therefore represents a system in which discretionary authority to impose restrictions may theoretically exist under general public health law, but without any HIV-specific operationalization or guidance. 

## Discussion

The earliest and most influential restrictive guidance on HIV-infected HCWs was issued by the U.S. Centers for Disease Control and Prevention (CDC) in 1991 [29]. Drafted in the aftermath of the highly publicized Florida dentist case, this guidance reflected profound uncertainty regarding transmission risks. HIV-positive HCWs were advised not to perform exposure-prone procedures (EPPs) or to seek advice from an expert review panel to determine which activities might be permissible. Although framed as precautionary, this approach effectively treated HIV infection itself as disqualifying, rather than assessing measurable transmission risk.

Building on this precedent, the United Kingdom’s Department of Health published HSG(93)40 in 1993 [30], the first comprehensive national policy specifically addressing HIV-positive HCWs. This policy introduced mandatory expert consultation, systematic patient notification (“look-back”) exercises, and the establishment of the UK Advisory Panel (UKAP). The approach was categorical: HIV-positive HCWs were entirely excluded from EPPs, and retrospective patient notification was required. These measures, though intended to protect patients, proved highly stigmatizing and professionally damaging.

Both the CDC (1991) [29] and UK (1993) [30] policies epitomize the fear-driven regulatory environment of the pre-HAART era, when HIV infection was widely regarded as fatal and effective viral suppression was unattainable. Regulatory decisions were therefore driven by theoretical risk rather than empirical evidence.

This paradigm shifted fundamentally with the introduction of highly active antiretroviral therapy (HAART) in 1996, following the landmark trials by Hammer et al. [1] and Gulick et al. [2], which demonstrated that combination antiretroviral therapy could suppress viral replication to undetectable levels. These advances transformed HIV into a manageable chronic condition and provided a virological basis for reassessing occupational transmission risk.

Subsequent guidelines abandoned blanket prohibitions in favor of risk-based, virologically informed approaches. Viral load emerged as the central regulatory parameter, replacing infection status as the determinant of professional fitness. Contemporary guidelines are therefore unified by the principle that sustained viral suppression eliminates measurable transmission risk, allowing HCWs to continue clinical practice under defined conditions. Despite this scientific consensus, however, national responses have evolved unevenly. As demonstrated by our review, practical implementation diverges markedly with respect to operational philosophy, governance structures, and degrees of professional autonomy.

Where explicit thresholds are defined, the German DVV/GfV 2012 guideline [11] stands out for its scientific rigor and operational clarity, applying the most conservative viral-load threshold (≤50 copies/mL), tolerating only short-lived low-level viraemia, and mandating quarterly monitoring under occupational health oversight. This contrasts with the broader threshold of <200 copies/mL adopted in Spain [14], the UK [13], France [15], Australia [20], Japan [12], and the United States [10].

A further axis of divergence concerns reinstatement to EPPs after loss of viral suppression. Spain, the UK and Australia apply explicit procedural requirements, mandating confirmation of renewed suppression through repeated testing over defined intervals before EPPs may be resumed. Australia represents the most algorithmic and surveillance-intensive model, with clearly specified escalation and reinstatement pathways. In contrast, the US, France, and Canada [21] rely on expert-led, case-by-case reassessment without fixed national confirmation intervals, while Germany anchors reinstatement to its strict numerical threshold without recourse to a national expert committee. These differences reflect not disagreement about transmission biology, but varying regulatory approaches to managing rare episodes of detectable viraemia.

Against this backdrop, the Swiss recommendations [15] appear increasingly anachronistic. By treating EPP participation as an exception requiring committee approval in the absence of an evidence-based viral-load threshold, they reverse the logic adopted by most contemporary frameworks and constrain professional autonomy without clear scientific justification.

Among European models, the Netherlands [17] and Denmark [18] represent the most liberal approaches. The Dutch framework relies entirely on the treating physician’s professional judgment, without numeric thresholds, expert panels, or reporting obligations. Denmark adopts an even more universalist stance, embedding HIV within a general hygiene-based infection-control framework that renders virological status irrelevant as long as standard precautions are observed. However, while these trust-based systems maximize professional autonomy and minimize stigma, their lack of formalized procedures may create uncertainty for institutions, insurers, and cross-border practice, and they depend heavily on professional integrity and ethical maturity.

In contrast, many EU member states have not articulated any HIV-specific framework for HCWs. In countries such as Hungary [25], Slovakia [26] and Italy [27], regulatory authority rests on vague or legacy provisions within general public health or occupational safety law, offering neither scientific specificity nor procedural clarity. Although undocumented local practices or unpublished institutional policies may exist, the persistence of regulatory silence or ambiguity reflects divergent governance philosophies rather than unresolved scientific questions.

## Conclusion

The absence of documented provider-to-patient HIV transmission in the era of effective antiretroviral therapy [31] underscores the need for greater international alignment. Replacing historically rooted or ambiguous frameworks with transparent, evidence-based standards would better safeguard both patient safety and the professional rights of HCWs living with HIV. Notably, the majority of contemporary guidelines that define a quantitative threshold converge on a viral load cut-off of <200 copies/mL as the operative standard for eligibility to perform EPPs. This convergence across multiple high-income countries suggests the emergence of an international de facto benchmark. However, systematic evaluations of the long-term outcomes and real-world performance of these threshold-based frameworks remain limited and should be prioritized in future research. In particular, the highly liberal, non-numerical approaches adopted in the Netherlands and Denmark are difficult to evaluate in the absence of structured monitoring or outcome data, underscoring the need for prospective assessments to determine whether different governance models achieve comparable levels of patient safety and legal certainty.

## Notes

### Authors’ ORCIDs

- Diel R: https://orcid.org/0000-0001-8304-7709
- Nienhaus A: https://orcid.org/0000-0003-1881-7302

### Competing interests

The authors declare that they have no competing interests.

## Figures and Tables

**Table 1:**
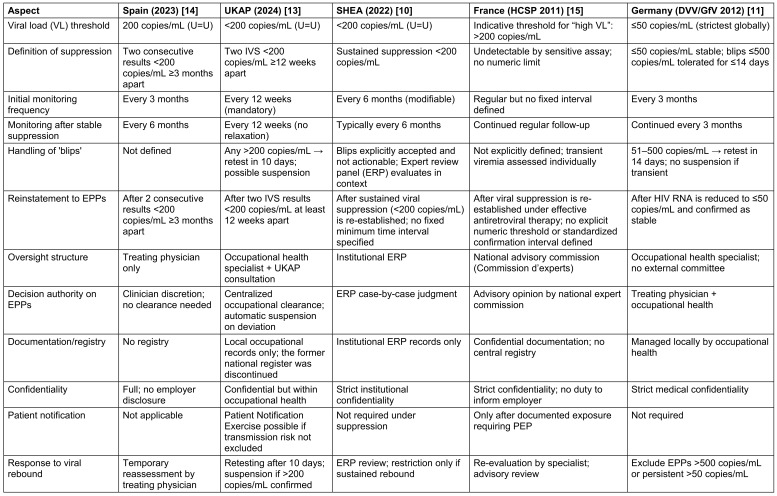
Comparison of HIV-infected HCW guidelines of the countries Spain, UK, USA, France, and Germany

**Table 2:**
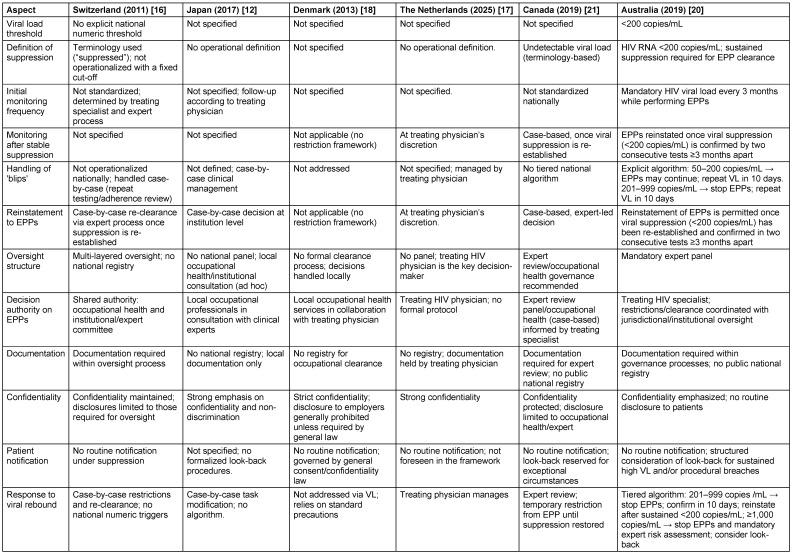
Comparison of HIV-infected HCW guidelines of the countries Switzerland, Japan, Denmark, The Netherlands, Canada, and Australia
